# The effects of nitrogen addition on dissolved carbon in boreal forest soils of northeastern China

**DOI:** 10.1038/s41598-019-44796-x

**Published:** 2019-06-04

**Authors:** Liang Shi, Jeffery P. Dech, Huaxia Yao, Pengwu Zhao, Yang Shu, Mei Zhou

**Affiliations:** 1 College of Grassland, Resources and Environment, Inner Mongolia Agricultural University, Hohhot, 010011 China; 2 Department of Biology and Chemistry, Nipissing University, North Bay, ON P1B 8L7 Canada; 3 Dorset Environmental Science Centre, Ontario Ministry of Environment and Climate Change, Ontario, P0A 1E0 Canada; 4 College of Forestry, Inner Mongolia Agricultural University, Hohhot, 010011 China

**Keywords:** Forest ecology, Ecosystem ecology

## Abstract

Understanding the effects of nitrogen (N) addition on dissolved carbon in boreal forest soils is essential for accurate evaluation of regional carbon balances. The objective of this study was to determine the effects of different levels and types of N addition on soil dissolved carbon concentration in a cold-temperate coniferous forest through an *in-situ* fertilization experiment. Simulated atmospheric N addition was applied in a factorial experiment with N addition level (control, 10, 20 and 40 kg of N ha^−1^yr^−1^) and N type (NH_4_Cl, KNO_3_ and NH_4_NO_3_) treatments. The experiment was conducted over the 2010 growing season (May-September) at the Kailaqi farm of Genhe Forestry Bureau, located in the northern Great Xin’an mountain range, northern China. Monthly N addition treatments were applied in three replicate plots per treatment (n = 36), and measurements of dissolved organic carbon (DOC) and dissolved inorganic carbon (DIC) were derived from monthly sampling of the organic and mineral soil horizons. There was a significant effect of N type, with the combined N source (NH_4_NO_3_) producing significantly higher DOC than the control (ambient addition) or the NH_4_Cl treatment in both the organic and mineral layers. The N addition treatment increased DIC in the organic layer at the low levels only, while N type did not have a significant effect. There was a significant interaction of the month and the N level treatment, as low level N addition tended to increase the content of soil DOC while high level N tended to inhibit soil DOC content, with these trends being most pronounced in the middle of the growing season. These results elucidate the importance of the type and timing of N additions to the dynamics of soil carbon pools.

## Introduction

The amount of atmospheric nitrogen (N) has increased considerably in recent decades as a result of higher anthropogenic emissions, resulting in significant changes in carbon and N cycles in terrestrial ecosystems^1,2^. The annual addition rate of reactive N in the atmosphere has increased from 15 Tg yr^−1^ in 1860 to 187 Tg yr^−1^ in 2005^1^, and it is expected that the rate will double in next 25 years^3^. China currently ranks as the region with the third highest N addition in the world following Europe and United States, with a mean total atmospheric dry and wet N deposition across the country of 12.9 kg ha^−1^yr^−1^, and values reaching up to 63.5 kg ha^−1^ yr^−1^ in some areas^4,5^. Increases in atmospheric N level affect processes such as carbon uptake, photosynthetic allocation, plant growth, litter decomposition, soil organic matter turnover and soil respiration^6^. Hence, increased N levels greatly interfere with forest ecosystem processes and functions^7^.

The dissolved organic carbon (DOC) is a component of the soil’s active organic carbon^8^, and the organic carbon source for microorganisms present in the soil. The DOC can be derived directly from litter, humus decomposition, precipitation input and fine root secretions^9^. In terrestrial ecosystems, DOC accounts for only a small fraction of C output^10^, but it plays an important role in the interactions between the C and N cycle. Specifically, C decomposition is coupled with N availability, dissolved organic N (DON) decomposition resembles that of DOC, and DOC decomposition is fueled by N mineralization^9^. Despite these known connections, the effects of increasing N deposition on soil DOC concentrations remain unclear. Reports have indicated that increased N levels either increase^11^, decrease, or do not affect^12^ the soil DOC concentration. Complicating the interpretation of these conflicting reports, the mechanism underlying the effect of N addition on soil DOC is still unknown.

Soil dissolved inorganic carbon (DIC) includes CO_2_, H_2_CO_3_, HCO_3_^−^ and CO_3_^2−^. These are compounds derived mainly from CO_2_ which reacts with water present in the soil to form H_2_CO_3_ and HCO_3_^−^. Since soil DIC content is much lower than soil DOC content, DIC is often overlooked in soil carbon balance studies. In addition, information on DIC dynamic responses to increasing N concentration are still needed^13^.

In recent years, studies on soil dissolved carbon and its dynamics have been conducted in forest, grassland, wetland and agricultural ecosystems^14,15^. These studies mainly focused on the influence of different management (e.g. tillage, fertilization) and land use practices on soil dissolved carbon content, distribution, leaching, migration, conversion and accumulation. Reports on the effects of N addition on soil dissolved carbon are rare. Moreover, the impact of N deposition on dissolved carbon in boreal coniferous forest ecosystems is unknown. The atmospheric N deposition in Great Xin’an mountain region is substantial, ranging from 9.87 to 14.25 kg N ha^−1^ yr^−1^ ^6^. Long-term inputs of low-level N will increase N availability in boreal forest ecosystems, affecting regional soil CO_2_ emissions and the soil carbon balance^16^. Although the mechanisms controlling the interaction of C and N in soils have not yet been fully understood, a possible response of DOC and DIC to changed N conditions can be expected based on several previous findings. For example, N addition increases microbial activity and metabolism, which in turn may change the DOC status in the soil^17^. Mineral N sometimes interacts with lignin, increasing the stability of soil organic matter, so mineral N additions could decrease DOC. Furthermore, a site-specific C: N ratio may exist for the organic layer or other soil layers in a given ecosystem, which may influence the DOC/DIC responses to N changes^18,19^. Therefore, more experimental studies are needed to help clarify the effects and interactions of different N addition scenarios. The objective of this study was to determine the effects of different levels and types of N addition on soil dissolved carbon concentration in a cold-temperate coniferous forest through an *in-situ* fertilization experiment. We hypothesized that the type and level of nitrogen addition would significantly affect the soil dissolved organic carbon. We anticipate that our results will provide new insights into forest soil carbon dynamics in the context of N addition in northern forest ecosystems.

## Results

### Response of soil DIC and DOC to N addition

**Figure 1:**
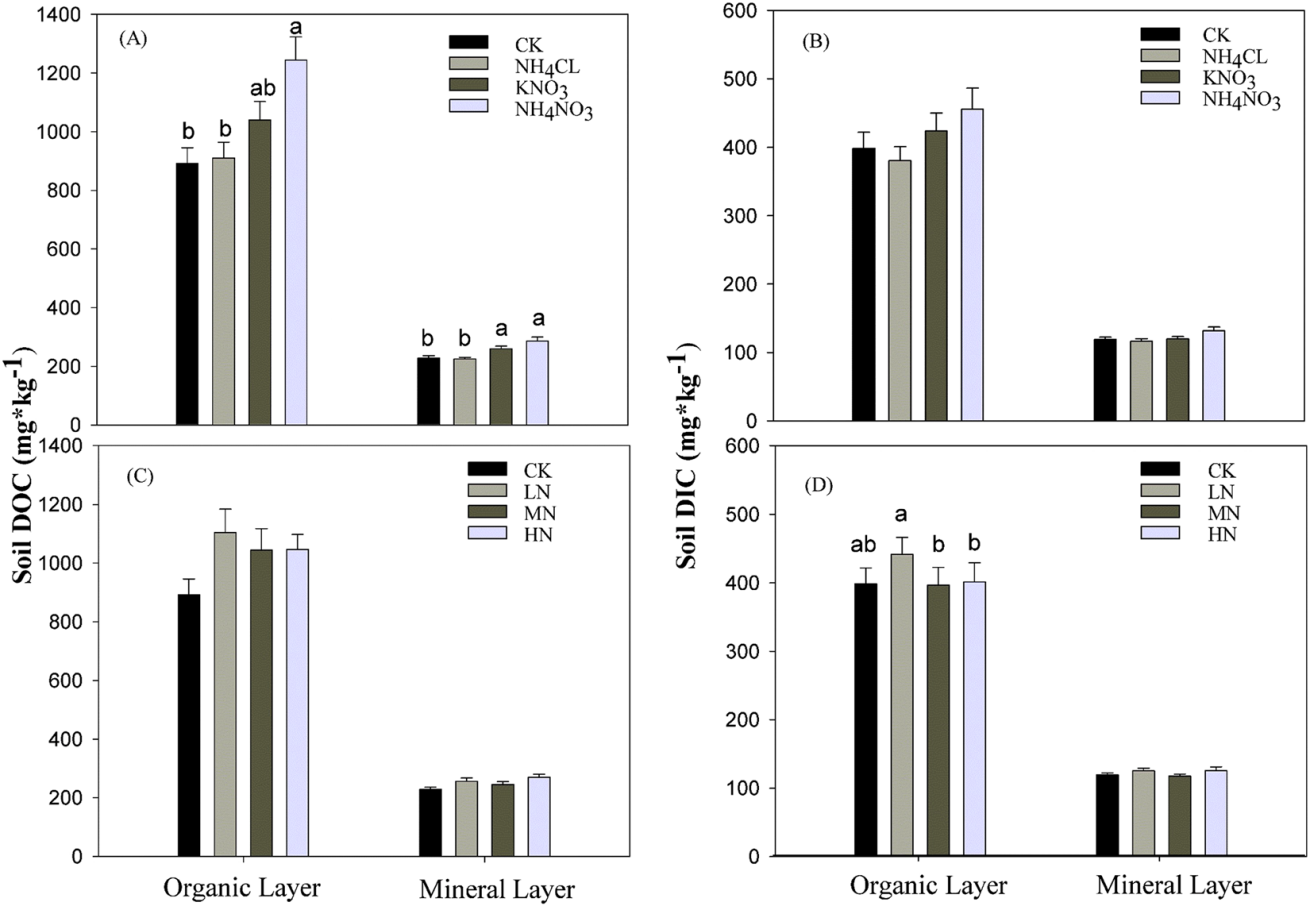
Mean ± 1 Std. Dev. of Soil DOC or DIC content vs soil layer (organic or mineral) for different N addition type treatments (**A,B**) and different N addition level treatments (**C,D**). LN means low = level N addition, MN = middle-level N addition, and HN = high-level N addition. Different lowercase letters indicate significant differences among treatment. Data collected over the 2010 growing season (May-September) at the Kailaqi farm of Genhe Forestry Bureau, located in the northern Great Xin’an mountain range, northern China.

Under ambient N addition conditions represented by the control treatment, the average DOC across all five months in the organic layer was 891.50 mg·kg^−1^, 390.08% greater than that of the mineral soil layer (Fig. 1). The highest DOC occurred in the combined NH_4_NO_3_ and KNO_3_ N addition type treatments (Fig. 1A). There were significant differences in soil DOC concentrations among some of the different N addition type treatments in both the organic and mineral layers (Table 1). In both layers we observed that the NH_4_Cl treatment was not significantly different than the control. In the organic layer, the NH_4_NO_3_ treatment produced significantly higher DOC values than the control or the NH_4_Cl treatment; however, there was no difference between the NH_4_NO_3_ and the KNO_3_ treatments (Fig. 1A). In the mineral layer, the NH_4_NO_3_ and the KNO_3_ treatment produced significantly higher DOC than the control or the NH_4_Cl (Fig. 1A). Again, there was no difference between the NH_4_NO_3_ and KNO_3_ fertilizers. Soil DOC was slightly higher in all N addition level treatments in comparison to the control (Fig. 1C). However, there were no significant difference among the control or any of the N addition levels for either the organic or mineral layers (Table 1).

**Table 1:** F statistics and p estimates for repeated measures analysis of variance on soil dissolved organic carbon, dissolved inorganic carbon and pH with N addition level and N addition type as between subjects effects and month as a within subjects effect.

*Source of variation*	*Soil DOC content*					*Soil DIC content*				*Soil pH value*	
	DF	F*-O layer*	P*-O layer*	F*-M layer*	P*-M layer*	F*-O layer*	P*-O layer*	F*-M layer*	P*-M layer*	F-M *layer*	P-M *Layer*
**Between subjects**											
N level	2	0.994	0.384	0.242	0.787	3.633	**0.041**	1.268	0.299	3.360	**0.041**
N type	2	4.828	**0.017**	4.301	**0.025**	2.317	0.119	0.339	0.715	0.541	0.589
N level × N type	4	1.605	0.204	1.578	0.211	1.611	0.203	0.722	0.585	0.730	0.580
**Within subjects**											
Month	4	6.127	**0.001**	1.117	0.353	54.233	**0.001**	37.587	**0.001**	1.061	0.313
Month × N level	8	2.273	**0.028**	1.051	0.403	1.204	0.304	1.022	0.425	0.488	0.620
Month × N type	8	0.482	0.867	1.176	0.321	0.577	0.795	0.794	0.609	0.846	0.441
Month × N level × N type	16	0.779	0.705	1.116	0.351	0.783	0.701	0.889	0.583	0.625	0.649

Data collected over the 2010 growing season (May-September) at the Kailaqi farm of Genhe Forestry Bureau, located in the northern Great Xin’an mountain range, northern China.

Notes: Values that are bolded represent statistical significance at p < 0.05.

Under control conditions, the average DIC across all five months from the organic layer was 398.52 mg·kg^−1^, which was 334.55% higher than the mineral soil layer (Fig. 1). N addition type had no significant effects on DIC in either the organic or the mineral soil (Fig. 1B, Table 2). The response of soil DIC to N addition levels was different than that of soil DOC, demonstrating a reduction with increasing N deposition. In organic layers, the soil DIC content was significantly higher in the low N addition level treatment compared to the medium and high N treatments. There was no difference between the low N addition level treatment and the control. In mineral layers, no significant differences were found (Fig. 1D).

**Table 2:** Stand characteristics and surface soil (0–20 cm) properties the study site.

Variable	
Location	50°56′N, 121°30′E
Mean tree age (year)	180
Elevation (m)	810
Net N mineralization (kg N ha^−1^yr^−1^)	71.7
Biomass (Mg C ha^−1^)	56.1 (4.8)
Gravel (0.2–2 mm, %)	51.76
Sand (0.002–0.02 mm, %)	27.55
Clay (0.002 mm, %)	9.53

Data collected over the 2010 growing season (May-September) at the Kailaqi farm of Genhe Forestry Bureau, located in the northern Great Xin’an mountain range, northern China.

Data source: Chinese Ecosystem Research Network (CERN) database. 10.1371/journal.pone.0089322.t00111/13/2018.

**Figure 2:**
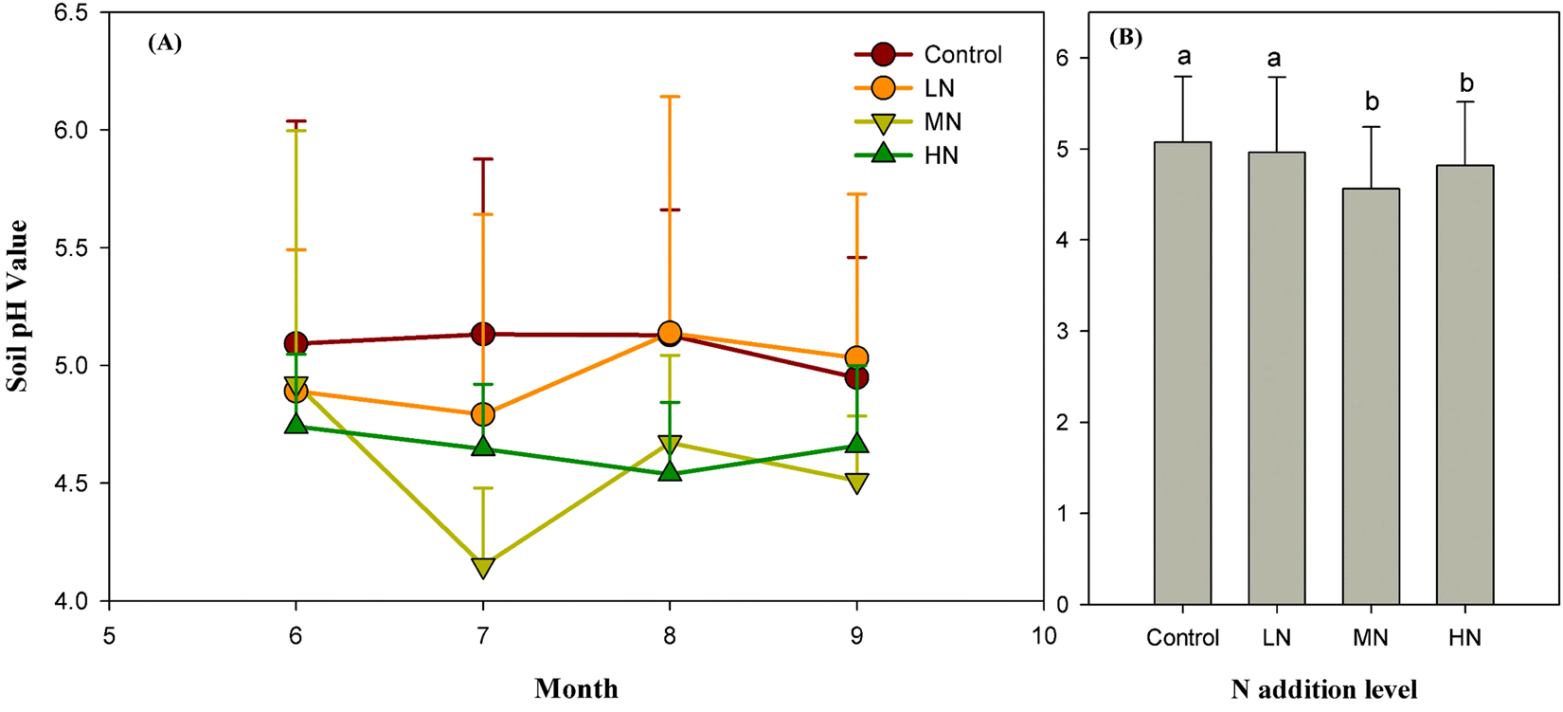
(**A**) Monthly variations of mean ± 1 std. dev. of Soil pH value under the different N addition level treatments of the mineral layer during the growing season (Jun to September) of 2010. LN = low-level N addition, MN = middle-level N addition and HN = high-level N addition (n = 9). (**B**) Average value of soil pH value for all months under different N addition levels. Different lowercase letters indicate significant differences among N addition levels. Data collected over the 2010 growing season (Jun-September) at the Kailaqi farm of Genhe Forestry Bureau, located in the northern Great Xin’an mountain range, northern China.

Only the N addition level significantly changed soil pH value (Table 1). High and medium N addition levels had significantly lower soil pH. The pH of soils in the Low N addition level treatment was not significantly different than the control (Fig. 2). The N addition type had no significant effects on soil pH.

### Monthly changes of the effects of N input

**Figure 3:**
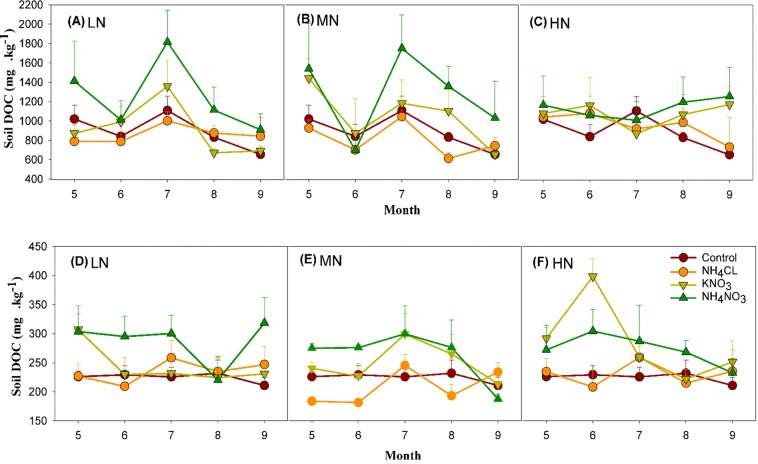
Monthly variations of mean ± 1 std. dev. of DOC under the different N type and N level treatments of the organic layer (**A–C**) and mineral layer (**D–F**) during the growing season (May to September) of 2010. LN = low-level N addition, MN = middle-level N addition and HN = high-level N addition (n = 3). Data collected over the 2010 growing season (May-September) at the Kailaqi farm of Genhe Forestry Bureau, located in the northern Great Xin’an mountain range, northern China.

Under control conditions, there was clear monthly variation in the DOC of the organic layer, with the peak amount occurring in July and the low in September. The DOC levels in the mineral soil were relatively constant throughout the season (Fig. 3). In most cases, the N addition treatments did not change the overall pattern of monthly changes in DOC compared to the control group. These Monthly fluctuations in soil DOC resulted in significant month effects for both organic and mineral layers (Table 1). In the organic layer, the DOC content was significantly different for pairwise comparisons of every month, except between May and July. The only significant two-way interaction was observed between month and N addition level treatment in the organic layer. This interaction was manifest as reduced monthly variation in DOC at the highest N additional level, which was significantly different than the low N addition level treatment (Fig. 3). No significant three-way interaction was found in DOC among the N-addition level, type, and month in either the organic or mineral layer of the soil (p > 0.05, Table 2).

**Figure 4:**
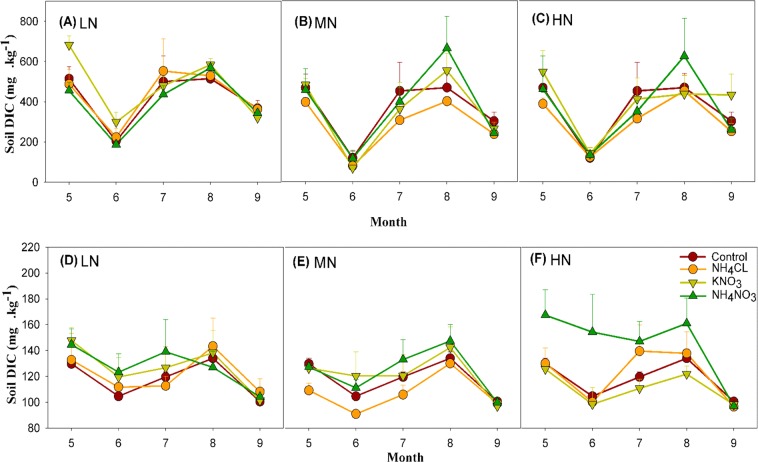
Monthly variations of mean ± 1 std. dev. of DIC under the different N type and N level treatments of the organic layer (**A–C**) and mineral layer (**D–F**) during the growing season (May to September) of 2010. LN = low-level N addition, MN = middle-level N addition and HN = high-level N addition (n = 3). Data collected over the 2010 growing season (May-September) at the Kailaqi farm of Genhe Forestry Bureau, located in the northern Great Xin’an mountain range, northern China.

Monthly variation in the DIC of the control followed a similar trend in both organic and mineral layers, with a clear peak in August and a low in June for the organic layer, and a peak in August and a slightly lower minimum in September rather than June for the mineral layer (Fig. 4). Similar to DOC, the differences in DIC content had significant monthly variations; however, these were significant in both organic and mineral layers (Table 2). There were no significant two-way interactions affecting the monthly variation of DIC. Furthermore, the three-way interaction of N addition level, type, and month on DIC was not significant in either the organic and mineral layer of the soil (Table 2). During the growing season, N addition decreased soil pH by 0.1–1.1 units. However, no significant difference in soil pH was observed among the different months.

## Discussion

The balance of soil carbon pools is mainly affected by the input from plants and the loss from leaching of active carbon components, such as DOC, DIC or the release of CO_2_ ^20^. In this study we reported that N addition significantly increased the concentration of the soil DOC. The type of N addition has a significant effect (p < 0.05); but the level of N addition has no significant effect (p > 0.05). Specifically, N addition in the form of nitrate generally affected soil DOC content in a stronger way than ammonium N addition.

In coniferous forest systems, the DOC content in the soil is mainly affected by litter degradation and rhizosphere deposition, although degradation of humus may also increase the percentage of soil DOC^21^. However, N addition can increase above-ground biomass and subsequent litter return^22^. At same time, N form is a restrictive condition, and the plants and soil microbes have a preference for NO_3_^−^ ^23,24^. During the growing season, plants and microbes typically use more NO_3_^−^ than other forms of available N^25,26^. Therefore, our results can be interpreted as further evidence to suggest that plants and microbes absorbed higher amounts of N in the form of NO_3_^−^ from the soil during the experiment. For humus-rich topsoil, increased microbial metabolic activity will promote soil DOC release. The fact that the soil DOC content increased even under the increased microbial activity in presence of nitrate N is notable^23^, given that the treatments appeared to increase substrate utilization and alter the soil biota activity and composition.

It is also possible that alternative microbial mechanisms^27^ may explain this pattern. Hagedorn^28^ suggests that experimental N addition changes the physical and chemical adsorption capacity of mineral soil organic matter due to increasing ionic strength, soil N availability and decreasing soil pH. In our study, since application of high level nitrate led to soil acidification (Fig. 2), the H^+^ increase would make the original equilibrium reaction shift to the next reaction step (H^+^ + HCO_3_^−^ ≒ 2H^+^  + CO_3_^2−^ ≒ H_2_O + CO_2_), potentially increasing soil CO_2_ emissions^29^. Sodium nitrate addition consistently increases DOC, whereas ammonium salts additions usually decreases DOC^30^. We also reported that three levels of N input slightly increased DOC content in the organic and mineral soil layer compared to the control condition; however, the differences were not statistically significant.

**Figure 5:**
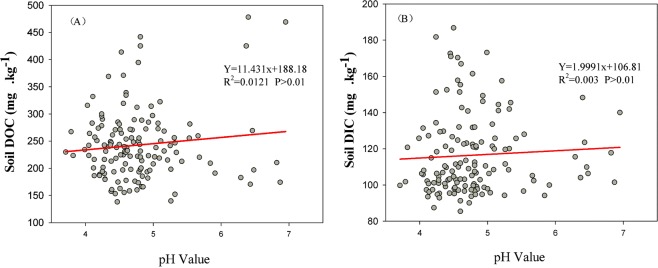
The relationship between the pH value and (**A**) DOC and (**B**) DIC. Red lines show the linear relationships tested; the R^2^- and P-values indicate the fitted regression coefficients and their significance, respectively. Data collected over the 2010 growing season (May-September) at the Kailaqi farm of Genhe Forestry Bureau, located in the northern Great Xin’an mountain range, northern China.

We found that the effect of different N levels on the DIC content of soil organic layer was consistent with the change of DOC; however, different N addition types had no significant influence on soil DIC content. In the case of DIC, the N level effect produced significant differences, and the promotion effect was the strongest at low N levels. High-level N addition likely increased the microbial utilization of soil DIC and accelerated DIC decomposition. Alternatively, soil acidification caused by high levels of N addition may have caused release and dissolution of inorganic carbon^31^. Previous studies have shown that in the boreal forest, where soil pH is acidic, soil inorganic carbon changes very little^32^. However, in our study, it was found that in the initial progress of N addition, soil acidification did not have a significant effect on soil DOC and DIC content (Fig. 5). Continued N treatment would result in significant soil acidification. However, in the initial stages of increasing N addition as represented in our experiment extending over a single growing season, it appears that the absorption of inorganic N by roots and microorganisms and the buffering capacity of the soil itself alleviated the effect of soil acidification on the DOC and DIC content to some extent^33,34^. Changes in soil DIC and DOC content could also be affected by soil CO_2_ flux. Because soil DIC content is much lower than soil DOC, the DIC dynamics have often been ignored in previous studies of soil carbon chemical balance.

Under most conditions, the addition of N did not change the overall pattern of monthly changes in soil DOC and DIC as compared to the control. In many studies only minor or no effects of addition N have been found^35,36^. We found that the soil DOC changes significantly and rapidly between months. This is because Soil DOC is affected by multiple environmental variables such as temperature, soil pH and nutrient supply. Water availability and temperatures which are directly influenced by monthly variations in weather, have close relationships with the DOC content^37,38^. We observed that the DOC responses to N input are stronger in July than September, which might be associated with temperature. With warmer temperatures, interactions with root-associated microorganisms, and root activity may increase^39^. Rise in temperature leads to accelerated plant litter formation, which is converted into organic matter^40^, and subsequently, soil DOC content increases. During September, microbial activity decreases due to the fall in temperatures, which ultimately reduces soil DOC content^41^. N addition may influence this pattern by promoting plant root growth and carbon distribution through roots, which in turn may change the soil DOC content. As the roots mature, the carbon assimilation and distribution by roots may also decrease, thus decreasing soil DOC content^42^. Prechtel *et al*.^43^ related monthly changes of soil DOC concentration to changes in associated microbial activities, suggesting that these might also be driving temporal patterns of DOC. In a forest setting, this physiological complexity is compounded by the variability inherent in many ecosystems, where large changes in soil characteristics frequently occur over short distances and short spans of time^44^.

According to our observations, the only significant two-way interaction was observed between month and N addition level treatment in the organic layer. Low level N addition tended to increase the content of soil DOC whereas high level N tended to inhibit soil DOC content, and this pattern was especially apparent in the middle of the growing season. It is interesting that DOC at high levels of N addition is reduced. Generally, DOC mainly derives from rhizo-deposition and litter degradation^45^. Previous research has suggested that high-level N addition increased the microbial utilization of soil DOC, accelerated DOC decomposition, and promoted the process of soil CO_2_ emission considerably^22^. Thus, N fertilizer application could increase DOC over short time scales, but after DOC achieves stability, N fertilizer addition cannot further improve soil DOC content^46^. All of these processes could have dampened the response of DOC to increased N levels, and resulted in the non-significant change from the control or slight decrease in soil DOC in the high compared to the low-level N treatment.

## Conclusions

In summary, our results show that addition of N fertilizer treatments did not change the overall pattern of Monthly changes in DOC and DIC. When considering the separate effects of N addition types and levels, respectively, we found that type of N addition has a significant effect on soil DOC, whereas N addition levels have a significant effect on the soil DIC. The only significant two-way interaction was observed between month and N addition level treatment in the organic layer. This interaction was manifest as reduced Monthly variation in DOC at the highest N addition level, which was significantly different from the low N treatment. Although our study has provided some insights into the patterns of soil C with N addition in a boreal conifer forest ecosystem, gaps remain in our understanding of the linkage between microbial community dynamics and soil functions.

## Materials and Methods

### Site description

**Figure 6:**
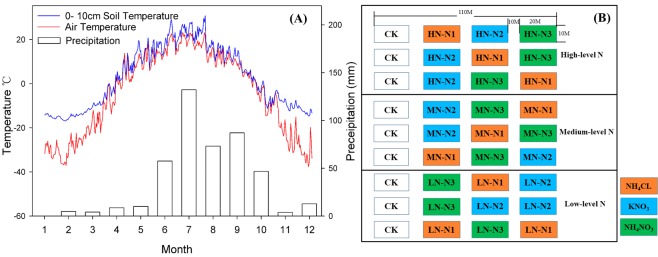
Air temperature, soil temperature (10 cm depth) and average precipitation in the study area during 2010 (**A**). Distribution of N addition experiment plots in the Great Xin’an mountain coniferous forest region. (CK = Control, N_1_ = NH_4_CL, N_2_ = KNO_3_, N_3_ = NH_4_NO_3_) (**B**).

The study was conducted at the Kailaqi farm of Genhe Forestry Bureau (51°00′N, 122°03′E), located in the Great Xin’an mountain range, northern China. The study site is in the southern edge of the boreal temperate permafrost zone. This area is characterized as a cold-temperate semi-humid climate, with a mean average annual temperature of −5.4 °C. About 60% of its annual precipitation (550 mm) occurs in July and August^47^. The snowfall period extends from the end of September to early May, with a mean snowpack thickness of 20–40 cm. Snowfall accounts for 12% of total annual precipitation^48^. The amount of surface evaporation is 800–1200 mm, with an average annual sunshine duration of 2594 hours and a frost-free period of 80 days (Fig. 6A). Soil is brown coniferous forest soil developed on granitic parent material. A mineral soil layer with thickness of 20 to 30 cm underlies a thick, well-developed organic layer. At our specific study site, the soil was generally shallow ranging from 40–50 cm in depth. The pH of soil water ranged from 4.5 to 6.5. Vegetation types were mainly Ledum - larch forest, with mean forest age of about 150a^49^. The dominant tree species were Xing’an larch (*Larix gmelini*), white birch (*Betula platyphylla*), with understory species including safflower Pyrola (*Pyrola incarnata*), Ledum (*Ledum palustre*) and red beans cranberry (*Vaccinium Vitisidaea*). A complete description of the site is given in Table 2.

### Experimental design

In order to simulate the effects of atmospheric deposition of NH_4_^+^, NO_3_^−^ as well as their combined effects, three N addition type treatments were used in this study, NH_4_Cl, KNO_3_, and NH_4_NO_3_. Based on the measured atmospheric N deposition flux at the study area (8.5 kg N ha^−1^yr^−1^), we designed four N addition level treatments representing different fertilizer input: (Control), which meant no N in addition to ambient background levels, low level N (Low-N, 10 kg N ha^−1^yr^−1^) which was a small increase to the *in-situ* addition level, medium level N (Medium-N, 20 kg N ha^−1^yr^−1^), and high level N (High-N 40 kg N ha^−1^yr^−1^). The last two fertilization treatments simulated realistic future atmospheric N deposition scenarios^50^. The amount of N input in last three treatments increased approximately 1.25, 2.0 and 4.0 times compared to the control treatment, respectively. Three replicate plots were established for each combination of N addition type (3) and N addition level (4) for a total of 36 plots (Fig. 6B). Each replicate plot was a 20 m × 10 m rectangle, and each plot in the experiment was spaced 10 m apart. During the 2010 growing season (May to September), N fertilizer was applied in each treatment by dissolving the appropriate level and type of fertilizer in 20 L of water, which was sprayed evenly on each of the plots at the beginning of each month. The control plots were sprayed with the same volume of water without fertilizer, to maintain a consistent watering regime. The experiment followed a common design employed in fertilization studies as outlined by Gao and others^51^. This work was conducted based on Forestry Standards “Observation Methodology for Long-term Forest Ecosystem Research” of People’s Republic of China.

### Soil sampling and analysis

At the mid-point of each month of the growing season, soil samples were collected from the organic and mineral layer in all experiment plots. For monthly collection in each plot, 10 sampling locations were randomly selected along a diagonal transect, and sub-samples were collected at each location from the organic layer on the top of the soil profile and the mineral layer at 0–10 cm depth using a 2.5 cm diameter soil auger. The 10 samples from each layer were subsequently mixed into one composite sample per plot per month for the organic and mineral layers, respectively.

We used a 2 mm soil sieve to remove gravel and plant roots immediately, and stored soil samples in a refrigerator at 4 °C before analysis. For DOC and DIC concentration analyses, we measured 15 grams of fresh soil from each sample using a 1/1000 analytical scale balance. We then added 100 ml of deionized water to the soil sample and oscillated the mixture for an hour before filtering it through a 0.45 μm membrane filtration unit. The soil DIC and DOC concentrations were analyzed using a Total Organic Carbon Analyzer (Liquitoc2 Elementar, Germany; detecting inorganic, organic and total carbons in a solution), and calculated according to the following equations:

1 $${\rm{DOC}}={\rm{DDOC}}\times 0.1\div{\rm{M}}\times (1-{\rm{SWC}})$$

2 $${\rm{DIC}}={\rm{DDIC}}\times 0.1\div{\rm{M}}\times (1-{\rm{SWC}})$$

where DOC and DIC are DOC and DIC concentrations (mg/kg) respectively for the sampled soil, DDOC and DDIC are filtrate DOC and DIC concentrations (mg/L) obtained by the analyzer, M represents fresh soil weight in grams (g), and SWC is the soil water content (%). Soil pH was measured using a pH meter (Mettler Toledo, Switzerland) and the ratio of soil to water was 1/2.5.

### Statistical analysis

Repeated measures analysis of variance (ANOVA) models were applied to examine mean differences in soil dissolved organic carbon, dissolved inorganic carbon and pH, with N addition level and N addition type as between subjects effects, and month as a within subjects effect. The ANOVAs were followed by a Tukey-HSD post-hoc tests to identify significant mean differences for each response variable. All statistical analyses were conducted using SPSS version 24.0 (IBM Corp., Armonk, NY, USA) and SigmaPlot version 12.5 (Systat Software, San Jose, CA). The level of statistical significance was set at p < 0.05.
